# The Effects of Incorporating Nanoclay in NVCL-NIPAm Hydrogels on Swelling Behaviours and Mechanical Properties

**DOI:** 10.3390/nano14070597

**Published:** 2024-03-28

**Authors:** Billy Shu Hieng Tie, Eyman Manaf, Elaine Halligan, Shuo Zhuo, Gavin Keane, Joseph Geever, Luke Geever

**Affiliations:** 1 Polymer, Recycling, Industrial, Sustainability and Manufacturing (PRISM) Centre, Technological University of the Shannon, Midlands Midwest, N37 HD68 Athlone, Ireland; a00218425@student.tus.ie (E.H.); a00238048@student.ait.ie (S.Z.); 2 Department of Mechanical, Polymer Engineering & Design, Technological University of the Shannon, Midlands Midwest, N37 HD68 Athlone, Ireland; eyman.research@gmail.com (E.M.); joseph.geever@tus.ie (J.G.); 3 Centre for Industrial Service & Design, Technological University of the Shannon, Midlands Midwest, N37 HD68 Athlone, Ireland; gavin.keane@tus.ie; 4 Applied Polymer Technologies Gateway, Materials Research Institute, Technological University of the Shannon, Midlands Midwest, N37 HD68 Athlone, Ireland

**Keywords:** hydrogels, smart materials, temperature-responsive, photopolymerisation, nanoclay

## Abstract

Following the formulation development from a previous study utilising N-vinylcaprolactam (NVCL) and N-isopropylacrylamide (NIPAm) as monomers, poly(ethylene glycol) dimethacrylate (PEGDMA) as a chemical crosslinker, and Irgacure 2959 as photoinitiator, nanoclay (NC) is now incorporated into the selected formulation for enhanced mechanical performance and swelling ability. In this research, two types of NC, hydrophilic bentonite nanoclay (NCB) and surface-modified nanoclay (NCSM) of several percentages, were included in the formulation. The prepared mixtures were photopolymerised, and the fabricated gels were characterised through Fourier transform infrared spectroscopy (FTIR), cloud-point measurements, ultraviolet (UV) spectroscopy, pulsatile swelling, rheological analysis, and scanning electron microscopy (SEM). Furthermore, the effect of swelling temperature, NC types, and NC concentration on the hydrogels’ swelling ratio was studied through a full-factorial design of experiment (DOE). The successful photopolymerised NC-incorporated NVCL-NIPAm hydrogels retained the same lower critical solution temperature (LCST) as previously. Rheological analysis and SEM described the improved mechanical strength and polymer orientation of gels with any NCB percentage and low NCSM percentage. Finally, the temperature displayed the most significant effect on the hydrogels’ swelling ability, followed by the NC types and NC concentration. Introducing NC to hydrogels could potentially make them suitable for applications that require good mechanical performance.

## 1. Introduction

In recent years, the discovery of smart materials has become popular in scientific research areas such as materials science and biomedical engineering [1]. Particularly in biomedical applications, stimuli-responsive hydrogels stand out as one of the most widely researched smart materials due to their unique reaction towards external stimuli. Conventional hydrogels swell by taking the water molecules into their polymeric network when immersed in water and de-swell when removed from water by repelling water molecules from their polymeric network [1,2]. However, stimuli-responsive hydrogels’ swelling behaviour is further controlled by the stimuli, including temperature [3], pH [4], magnetic [5], light [6], ultrasound [7], etc. This smart feature of stimuli-responsive hydrogels allows them to be utilised as customised solutions for problems such as wound treatment [4,8], drug release [7,9], antitumor [10,11], tissue engineering [12], and soft robots [13].

Poly(N-isopropylacrylamide) (PNIPAm) and poly(N-vinylcaprolactam) (PNVCL) as two of the most popular thermo-responsive hydrogels [14,15] exhibit outstanding swelling responsiveness to temperature. PNIPAm and PNVCL are negatively thermo-responsive hydrogels featuring a lower critical solution temperature (LCST), where they are soluble at temperatures below their LCST and insoluble at temperatures above their LCST [16]. This is the reason why negatively thermo-responsive hydrogels swell at temperatures lower than their LCST but de-swell at temperatures above their LCST. The close-to-physio LCST of both PNIPAm and PNVCL at 32 °C leads to increased interest in applying them in biomedical applications [14,15]. Previous studies have also proven that the LCST of hydrogels can be tuned by incorporating comonomers [17,18]. Moreover, the biocompatibility, biodegradability, swellability, and flexibility of hydrogels allow them to be developed into commercial products in biomedical applications [19]. 

Physically crosslinked hydrogels are weak and have weakened mechanical properties after swelling [20]. The ionic interactions between the polymer chains of physically crosslinked hydrogels lead to their poor mechanical properties and dissolution in water over time [2]. However, through chemical crosslinking, covalent bonds are formed between the polymer chains, which leads to enhanced mechanical properties and stability [21]. Also, by incorporating a chemical crosslinker into hydrogels, their swelling percentage is reduced [22]. The combination of PNIPAm and PNVCL was showcased previously with different concentrations of chemical crosslinker, and it was proven that higher crosslinker concentration can lead to lower swelling percentage and higher storage modulus [23].

Additionally, the mechanical properties of hydrogels play an important role in deciding their capability within several applications. For example, sufficiently strong hydrogels are required for mechanical loading in bone grafting [24]. Apart from incorporating crosslinkers to improve the mechanical properties of hydrogels, studies have shown that through incorporating NC, the mechanical properties of nanocomposite hydrogels are improved [25,26,27]. NCs are mineral silicate layers with a thickness of approximately 1 nm, and they are typically used for mechanical strength enhancement [28]. Through the incorporation, mutual physical interactions between the NC surface and polymer chains result in the NC composite hydrogels [27]. By introducing NC, the swelling behaviours and the mechanical properties of NVCL-NIPAm hydrogels could be enhanced. 

The aim of this research is to study the effects of NC incorporation on the swelling behaviours and mechanical properties of NVCL-NIPAm hydrogel that was formulated in a previous study [23]. FC34 has shown the capability to swell both below and above its LCST in three cycles of pulsatile swelling, indicating its potential application as a shapeshifting hydrogel structure, such as a thermosensitive bio-actuator. Although the formation of cracks was noticed after the pulsatile swelling, the incorporation of NC could potentially minimise the formation of cracks appearing due to the physical interaction between the polymer chains and NC. An improved understanding of the interactions between NCs and NVCL-NIPAm would further add to the design and development of smart materials with tailored characteristics, as well as the potential development of NC-incorporated NVCL-NIPAm-based hydrogels for biomedical applications. 

## 2. Materials and Methods

### 2.1. Materials

N-vinylcaprolactam (NVCL) (Mw = 139.19 g/mol; storage temperature 2–8 °C) was acquired from Sigma Aldrich Ireland (Dublin, Ireland). The second monomer, N-isopropylacrylamide (NIPAm) (Mw = 113.16 g/mol; storage temperature 0–10 °C), was procured from TCI Europe (Paris, France). Poly(ethylene glycol) dimethacrylate (PEGDMA) (Mw = 550 g/mol) was obtained from Sigma Aldrich Ireland (Dublin, Ireland), functioning as a chemical crosslinker. The photoinitiator, 4-(2-hydroxyethoxy) phenyl-(2-hydroxy-2-propyl) ketone (Igracure^®^ 2959 Ciba Corp., New York, NY, USA), was obtained from Ciba Specialty Chemicals (Basel, Switzerland). The additives, surface-modified nanoclay (NCSM) (particle size ≤ 20 µm) and bentonite nanoclay (NCB) (particle size ≤ 25 µm), were both purchased from Sigma Aldrich Ireland (Dublin, Ireland). Both types of NC supplied have montmorillonite, a type of smectite clay mineral, as their core mineral structure. The NCSM supplied contains 15–35 wt.% octadecylamine and 0.5–5 wt.% aminopropyltriethoxysilane that altered the NC surface properties. Table 1 provides the list of materials with their corresponding chemical structures or compositions.

### 2.2. Synthesis of Nanoclay-Incorporated Copolymers

The fabrication of NC-incorporated chemically crosslinked (Table 2) and physically crosslinked (Table 3) xerogels was completed using a UV irradiation chamber accordingly. This UV irradiation chamber (Dr. Gröbel UV-Elektronik GmbH, Ettlingen, Germany) establishes free-radical polymerisation through its built-in 20 UV tubes that provide an average UVA (315–400 nm) intensity of 10–13.5 mW/cm^2^. Prior to UV irradiation, NVCL was placed in a water bath at 50 °C for 1 h, and the formulations were blended in individual beakers using a magnetic stirrer at 150 rpm and 50 °C for 20 min until homogeneous. The homogenous mixtures were then pipetted into circular discs (diameter = 30 mm; thickness = 4 mm) with impression-containing silicone moulds for photopolymerisation. The photopolymerisation process was conducted for 10 min on each face of the circular discs. The cured circular discs were then dried in a vacuum oven at 50 °C for 24 h. 

#### Preparation of Aqueous Solutions

Fabricated physically crosslinked xerogels based on Table 3 were dissolved in distilled water at a ratio of 1:19 (gel/distilled water) to form aqueous solutions with a 5 wt% hydrogel concentration. The mixtures were kept at room temperature in capped flasks until the hydrogels were completely dissolved.

### 2.3. Design of Experiment with Swelling as the Response

The design of the experiment (DOE) was performed to investigate the relationship between multiple factors and hydrogel swelling behaviours. In this investigation, swelling temperature, types of NC, and percentages of NC were selected as factors. A triplicated full factorial DOE was conducted following the parameters shown in Table 4. Minitab 21.4.1 statistical software (Minitab^®^, State College, PA, USA) was used to analyse the data.

### 2.4. Attenuated Total Reflectance Fourier Transform Infrared Spectroscopy

Attenuated total reflectance Fourier transform infrared spectroscopy (ATR-FTIR) was conducted on a Thermo Scientific Nicolet iZ10 FTIR spectrometer (Thermo Fisher Scientific, Waltham, MA, USA) fitted with a universal ATR sampling accessory. All examinations were conducted at room temperature in the spectral range of 4000–650 cm^−1^, and the data were analysed using the Spectrum 10 software (Perkin Elmer, Waltham, MA, USA).

### 2.5. Lower Critical Solution Temperature Determination

#### 2.5.1. Cloud-Point Analysis

The prepared aqueous solutions were pipetted into individual test tubes, and these test tubes were immersed in a water bath with gradually increasing temperature for the measurement of cloud-point. In the Memmert water bath system (Memmert GmbH + Co. KG, Schwabach, Germany), an additional thermometer with an accuracy of +/−0.2 °C was employed for temperature monitoring. The temperature increased from 20 °C at a rate of 1 °C per 5 min until all solutions turned cloudy. 

#### 2.5.2. UV Spectroscopy

The LCST of aqueous solutions was further analysed using UV spectroscopy. A Synergy HT BioTek plate reader (BioTek, Winooski, VT, USA) fitted with a heating system was utilised. The optical absorbance at 500 nm was measured at every increment of 1 °C, starting from 20 °C to 50 °C. The 96-well plate was used and allowed to equilibrate for 20 min before each absorbance measurement.

### 2.6. Pulsatile Swelling Studies

In triplicate, the chemically crosslinked gels were placed into individual petri dishes (Thermo Fisher Scientific, Waltham, MA, USA) with a diameter of 90 mm and a height of 15.9 mm. All samples were weighed prior to the test, and 50 mL of distilled water was added to every petri dish. The swelling of the samples was initially performed at room temperature (approximately 20 °C), with the samples’ weight measured intermittently. When the sample weight became constant, the Petri dishes were transferred to a Salvislab oven (Salvislab, Rotkreuz, Switzerland), running at 50 °C until the constant weight of each sample was measured. The cycle was repeated three more times with the same samples.

The swelling ratio of the samples was calculated using Equation (1), where *W_t_* represents the weight of the swollen gels at a predetermined time, and *W_d_* represents the dry weight of the gels. During the weight-measuring procedure, the gel samples were removed from the water and blotted free of surface water using filter papers before being placed on the weighing scale.

(1) $$Swelling\;Ratio\;\left(\%\right)=\frac{W_{t}-W_{d}}{W_{d}}\times 100$$

On the other hand, gel samples used in the DOE were swollen to equilibrium at both 20 and 50 °C, using the same volume of distilled water. For rheological analysis, the gel samples swollen at 20 °C followed the same conditions.

### 2.7. Rheological Analysis

Fully swollen chemically crosslinked hydrogels at 20 °C were analysed using a Discovery HR30 rheometer (TA Instruments, New Castle, DE, USA). The rheometer was equipped with a stainless-steel Peltier plate and a temperature control system. A 20 mm diameter stainless-steel parallel plate was utilised for the amplitude sweep conducted at a frequency of 1 Hz, covering a strain range from 0.0001% to 10%. A constant axial force of 5 +/− 0.5 N was applied while performing all tests at a set temperature of 20 °C. A leather cutter of 20 mm diameter was used to prepare the rheological samples. The storage modulus of chemically crosslinked hydrogels was examined to investigate how the addition of NC affects their mechanical strength in the linear viscoelastic region.

### 2.8. Scanning Electron Microscopy

The dry gels were soaked in liquid nitrogen and subsequently fractured with a hammer to collect fragments for testing. The samples were imaged in a Tescan Mira 3 XMU Scanning Electron Microscope (Tescan, Brno, Czech Republic). All samples were gold-coated and imaged at 20 kV, and the SEM images were recorded at a magnification of 400×.

## 3. Results

### 3.1. Photopolymerisation

All samples were successfully fabricated through photopolymerisation, similar to our previous studies with NVCL-based hydrogels [17,18,23,29,30,31]. When compared to other polymerisation methods, the photopolymerisation method uses much less time and creates minimal waste of ingredients [32]. The key element for photopolymerisation to be successful is to include a light-sensitive ingredient, such as a photoinitiator, into the monomer mix before applying UV. Irgacure 2959 was the photoinitiator that absorbed the UV light to form free radicals, and these free radicals initiated the polymerisation of the monomer mix [33]. Furthermore, to expand the potential of applying the hydrogels in biomedicine, the use of a photoinitiator of low cytotoxicity was necessary. Williams et al. had previously reported that Irgacure 2959 is well tolerated by a range of mammalian cell types when compared to several photoinitiators [34].

**Figure 1:**
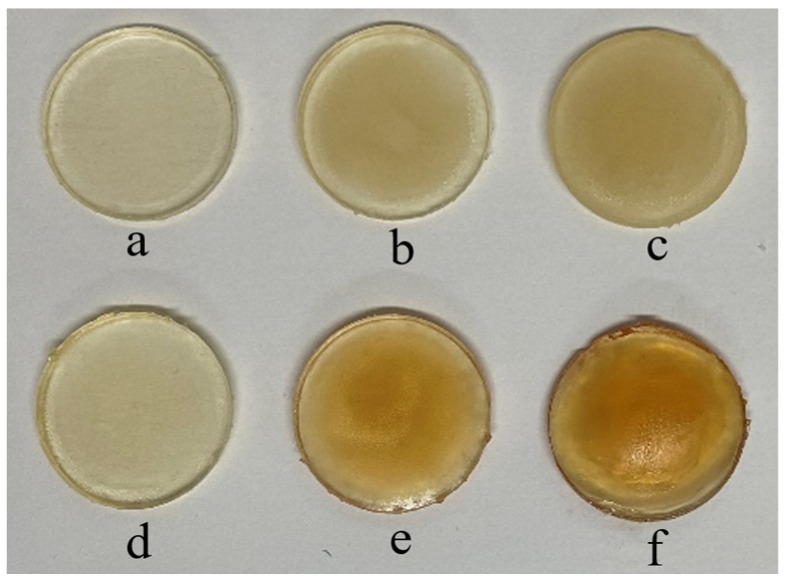
Photopolymerised chemically crosslinked gels containing (**a**) 0.1% NCB, (**b**) 1.0% NCB, (**c**) 3.0% NCB, (**d**) 0.1% NCSM, (**e**) 1.0% NCSM, and (**f**) 3.0% NCSM.

Both physically and chemically crosslinked gels were photopolymerised. The physically crosslinked gels were made to study their LCST using cloud-point analysis and UV spectroscopy, as chemically crosslinked gels do not dissolve in water. Moreover, the gels were crosslinked chemically to obtain tougher gels, as demonstrated in our previous work [23]. The composition of all ingredients remained the same so that the influence of incorporating different NCs with several concentrations could be investigated. According to the literature, the integration leads to mutual physical interactions between the surface of the NC and polymer chains, resulting in the formation of NC composite hydrogels [27]. The fabricated chemically crosslinked gels (Figure 1) were glass-like, and it was noticed that with higher concentrations of NCB and NCSM, the colour of the gels became darker. All samples were UV-cured for 10 min on each face of the disc to ensure consistency.

### 3.2. Design of Experiment with Swelling as the Response

DOE is a strategy used to obtain valid, reliable and sound conclusions when multiple variables influence a certain characteristic of a product [35]. In this study, chemically crosslinked gels were the product, and the variables influencing their swelling ratio were the types of NC, NC concentration, and swelling temperature. The swelling temperature typically has a significant effect on the hydrogels swelling ability. In our previous work with NVCL-based hydrogels, it was verified that hydrogels de-swell at temperatures above their LCST while maintaining a much smaller swollen percentage [17]. However, the effects of incorporating different types of NC and NC concentrations in FC34 are not yet known, nor is their significance when compared to the swelling temperature. Table 5 includes all the experiment runs with their corresponding response. The multi-level full-factorial DOE generated a total of 36 runs, with each set of replications consisting of 12 runs. Standard orders 1, 13, and 25, as well as 2, 14, and 26, etc., correspond to the experimental replicates. The DOE resulted in a regression model with an R2 value of 99.61%, as shown in Table A1. This suggests that 99.61% of the variation in swelling ratio is explainable by the three factors studied (types of NC, NC concentration, and swelling temperature). Moreover, the generated regression equation that predicts the hydrogel swelling ratio is shown in Equation (A1).

**Figure A1:**
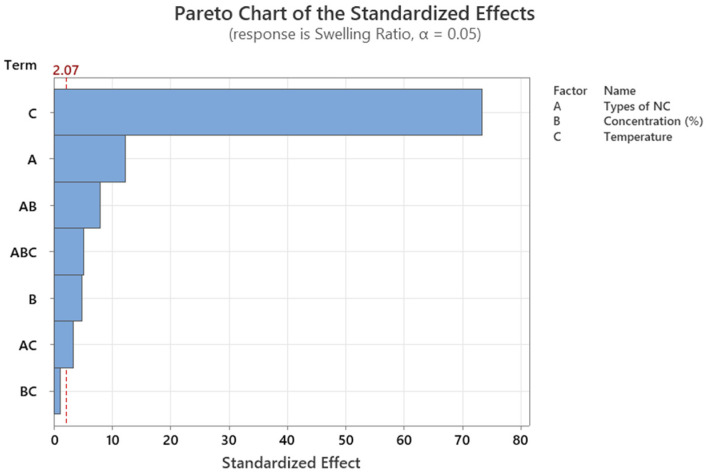
Pareto chart for the conducted DOE. The dashed line represents the reference line that is at 2.07, factor bars cross the reference line are statistically significant at the 0.05 level with the current model terms.

From the ANOVA table (Table A2), the linear terms (types of NC, NC concentration, and swelling temperature) are significant factors since their *p*-values are less than 0.05. Apart from the interaction between NC concentration and swelling temperature yielding a *p*-value of 0.253, the rest of the 2-way and 3-way interactions of variables are statistically significant with *p*-values less than 0.05. The Pareto chart in Figure A1 summarises all the statistically significant variables and interactions. The swelling temperature was found to have the largest effect on the hydrogels swelling ratio, while the types of NC had the second largest effect.

**Figure 2:**
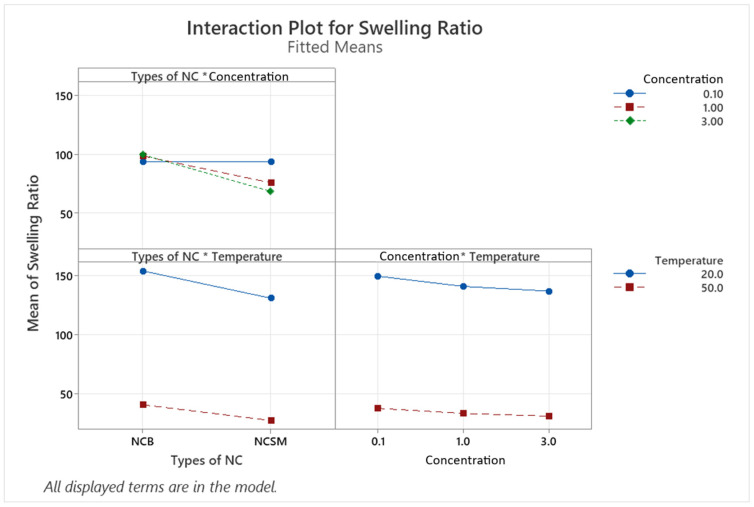
Interaction plot for the conducted DOE. Interaction terms are denoted by inserting an asterisk between the variables intended for interaction.

Examining the interaction plot in Figure 2, it can be observed that at 0.1% concentration, the types of NC did not have a noticeable effect on the swelling ratio. At both 1.0% and 3.0% concentrations, NCB results in higher swelling ratios than NCSM. In the interaction between the temperature and types of NC, going from a temperature of 20 °C to 50 °C results in lower swelling ratios for both types of NC, with NCSM exhibiting slightly lower swelling ratios as compared to NCB. Looking at the concentration interaction with temperature, higher NC concentrations at 1.0% and 3.0% have a slightly decreasing effect on the gels’ swelling ratio at both low and high temperatures. Yet, this interaction has been proven statistically insignificant by the ANOVA analysis.

### 3.3. Attenuated Total Reflectance Fourier-Transform Infrared Spectroscopy

**Figure 3:**
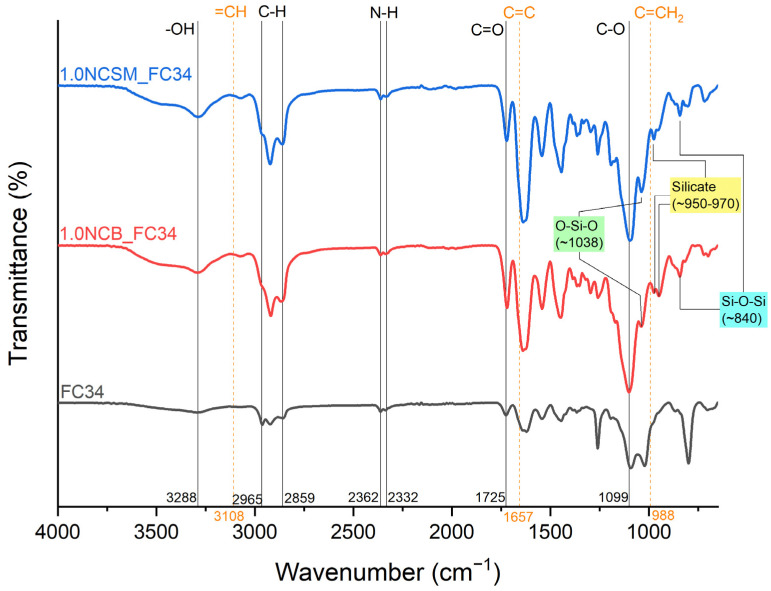
FTIR spectra of FC34, 1.0NCB_FC34, and 1.0NCSM_FC34.

ATR-FTIR is a popular tool for the immediate determination of mostly organic components, including chemical bonds and organic functional groups in both monomers and polymers [36]. ATR-FTIR was utilised in this study to investigate if the NC-incorporated chemically crosslinked gels were successfully polymerised via UV irradiation. Prior to the analysis, all tested samples were oven-dried at 50 °C for 24 h to ensure the leftover monomers were evaporated from the samples surface. Representative spectra are shown in Figure 3 containing FC34 as the control, and 1.0NCB_FC34 and 1.0NCSM_FC34 as the NC-incorporated samples. Successful polymerisation can be proven from the disappearance of =CH, C=C, and C=CH_2_ functional groups at 3108 cm^−1^, 1657 cm^−1^, and 988 cm^−1^, indicating the breakage of the double bond in monomers during the UV irradiation. The wavenumber at 3288 cm^−1^ represents the -OH functional group, confirming the hydrophilic nature of the gels absorbing moisture rapidly from the air after drying in the oven. Additionally, the C-H, N-H, C=O, and C-O bonding can be traced from the spectra at 2859–2965 cm^−1^, 2332–2362 cm^−1^, 1725 cm^−1^, and 1099 cm^−1^. The positive indication of NC present can be found in both NCB and NCSM-incorporated gels by the identification of O-Si-O, silicate, and Si-O-Si at 1038 cm^−1^, 950–970 cm^−1^, and 840 cm^−1^. The finding of these wavenumbers matches with the literature [36,37].

### 3.4. Lower Critical Solution Temperature Determination

**Figure 4:**
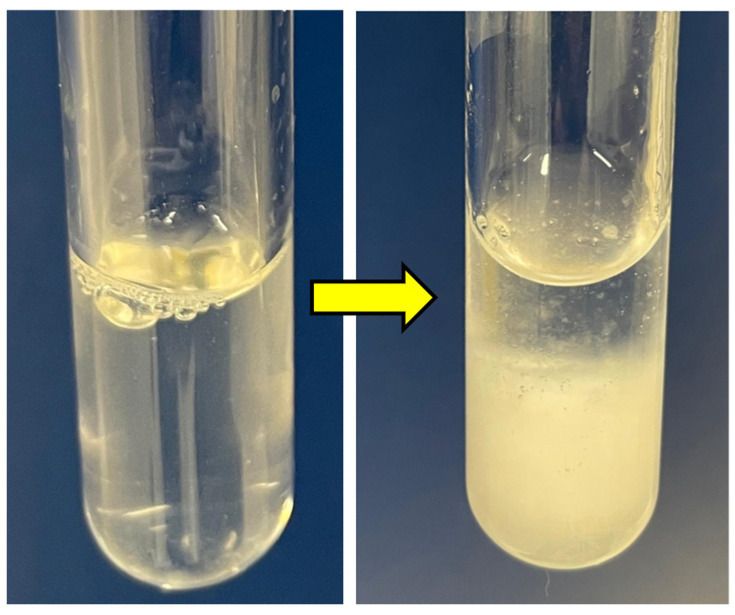
Transparent aqueous solution of 1.0NCSM_FP34 (**left**) became cloudy (**right**).

As negatively thermo-responsive hydrogels, both PNIPAm and PNVCL undergo a sol–gel transition in response to a change in temperature around their LCST [32,38]. In this phase transition, when the temperature reaches its LCST, the solution of the thermo-responsive hydrogels shifts to a gel state. At a temperature lower than the LCST, the existence of the hydrogen-bonding leads to the dissolution of polymer chains in water. During heating, energy is absorbed by the hydrogen bond, and when the LCST has been reached, the hydrogen bond breaks and the polymer chains shrink rapidly into the globular state, causing the precipitation of the polymer [16,39]. Apart from the sol–gel transition, the indication of reaching the LCST becomes recognisable when the solution changes appearance from transparent to cloudy (Figure 4). This has also been demonstrated in our previous study utilising the same base formulation [23]. However, unlike the usual observation of cloud-point that would show a cloudy solution of consistent appearance, the indication of LCST was shown with a phase separation of solution into a dilute phase and a concentrated phase, possibly due to the presence of NC pushing the system to partition.

**Figure 5:**
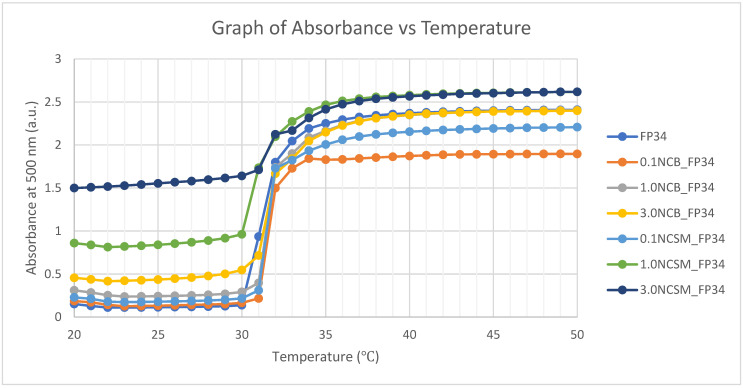
UV spectroscopy of the aqueous solutions applying a wavelength of 500 nm.

Hence, the LCST of each formulation was determined using both cloud-point analysis and UV spectroscopy. Both cloud-point analysis and UV spectroscopy recorded the onset temperature for the change in turbidity. This analysis resulted in a cloud-point temperature of 32 °C and 31 °C, resulting from the UV spectroscopy for all formulations (Table 6). The transition in turbidity of solutions was observed by the naked eye, which explains a slightly higher detected LCST when compared to the UV spectroscopy. In the UV spectroscopy (Figure 5), the absorbance of light at 500 nm was measured for every increment of temperature using the UV spectrophotometer, thus obtaining a more accurate LCST. The result indicated that the incorporation of a selected NC of 3% or less did not have significant effects on the NVCL-NIPAm hydrogel’s LCST. A total of 5 wt% of aqueous solutions were prepared, and as it was found out from a previous study, gels did not fully dissolve in distilled water with higher concentrations [23].

### 3.5. Pulsatile Swelling Studies

All the analysed samples had dry weights measured at 1.7 +/− 0.5 g initially. Pulsatile swelling was performed for all chemically crosslinked gels. Four and a half cycles were completed for each sample, with one cycle consisting of swelling to equilibrium at room temperature and subsequently at 50 °C. In applications requiring load bearing, the mechanical properties of materials are important so that they execute the assigned tasks without failing. However, many works have reported hydrogels having weakening mechanical properties as proportional to swelling [20,40]. FC34 was proven in a previous study to have completed three cycles of pulsatile swelling without falling apart, though cracks were observed throughout the sample [23].

**Figure 6:**
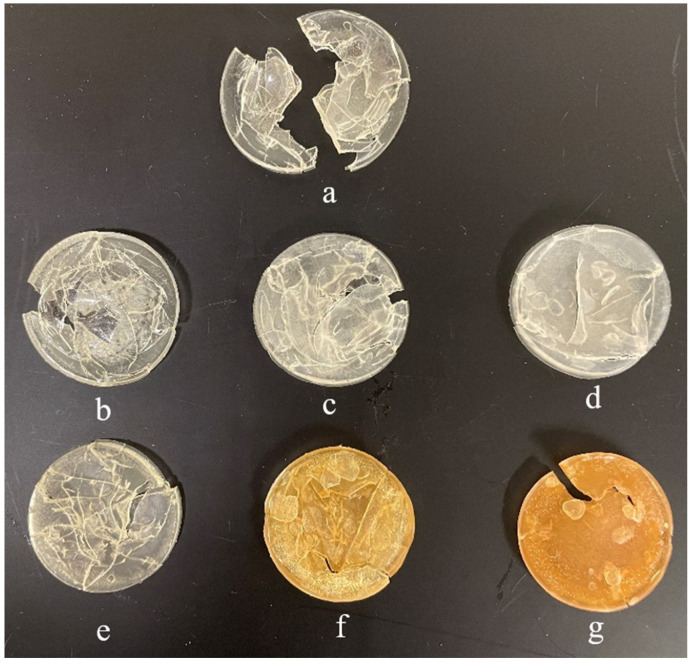
Dried gels after pulsatile swelling: (**a**) FC34, (**b**) 0.1NCB_FC34, (**c**) 1.0NCB_FC34, (**d**) 3.0NCB_FC34, (**e**) 0.1NCSM_FC34, (**f**) 1.0NCSM_FC34, and (**g**) 3.0NCSM_FC34.

In this study, NC-incorporated FC34 gels went through 4.5 cycles of pulsatile swelling, and all gels were then fully dried in an oven at 50 °C. The dried gels appearance is shown in Figure 6. It can be observed that FC34 gel broke in half after the pulsatile swelling. As compared to FC34, all other gels containing NC maintained their structural integrity with partial fracture, except 3.0NCB_FC34 gel. Thus, 3.0NCB_FC34 gel showed the best swelling integrity after 4.5 cycles of pulsatile swelling. Nonetheless, the formation of cracks in all gels was caused by the sudden internal stress formation during the rapid increase in water temperature above the LCST. At temperatures above the LCST, water molecules were swiftly expelled through the polymer hydrophobic segments, causing the shrinkage of polymer chains [41].

**Figure 7:**
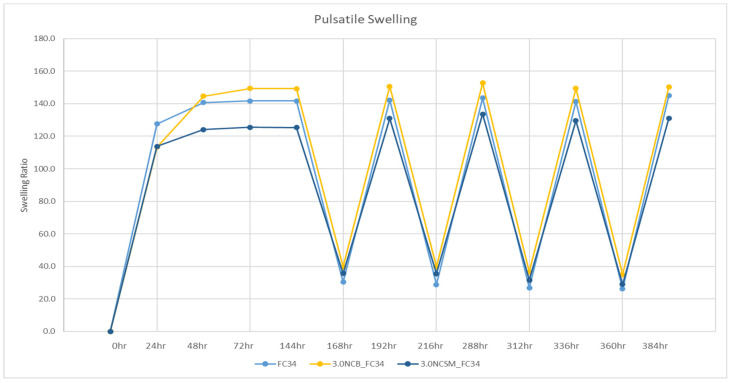
Pulsatile swelling curves of FC34, 3.0NCB_FC34, and 3.0NCSM_FC34. Swelling at room temperature including 0–144 h, 168–192 h, 216–288 h, 312–336 h, and 360–384 h; swelling at 50 °C including 144–168 h, 192–216 h, 288–312 h, and 336–360 h.

As analysed in the DOE, apart from swelling temperature, the types of NC showed the most significant effect on the swelling percentage of hydrogels. Hence, when compared to FC34 gels, NCB had an increasing effect on the hydrogels swelling percentage, but NCSM had a decreasing effect on the same. The pulsatile swelling results are compared in Figure 7, where the positive impact of NCB and the negative impact of NCSM on the gels swelling percentage can be clearly observed. Therefore, it indicates that NCB is a more suitable candidate than NCSM when incorporated in NVCL-NIPAm hydrogels, as it can further increase the hydrogels swelling ability by maintaining swelling integrity.

### 3.6. Rheological Analysis

The use of a rheometer to analyse the mechanical properties of hydrogels was demonstrated to be an effective comparative method [23,40]. However, it is challenging when it comes to clamping the swollen hydrogels as they usually deform, distort, and break under force before performing the analysis. Henceforth, a rheometer installed with a parallel plate, which causes minimal destruction to the hydrogels, was utilised. Storage modulus (G′) is the measure of stored energy, and stiff materials have large G′ [42]. A constant axial force of 5 +/− 0.5 N was applied to the samples while performing the analysis, which was higher than the previous parameter [23] since we have found out that under higher axial force, the slippage of gels against the plate can be minimised. In addition, an applied constant axial force was very beneficial in our situation, where all samples were of different thicknesses. The DOE could potentially include G′ as the second response, but a large number of materials would have been wasted during the cutting of gels to the suitable size for the rheological analysis, as gels at different temperatures possess different toughness and fragilities that could cause breakage easily. The standard method of cutting gels for the rheological analysis needs to be developed instead of using the leather cutter to improve the consistency and quality of the rheological samples prepared.

**Figure 8:**
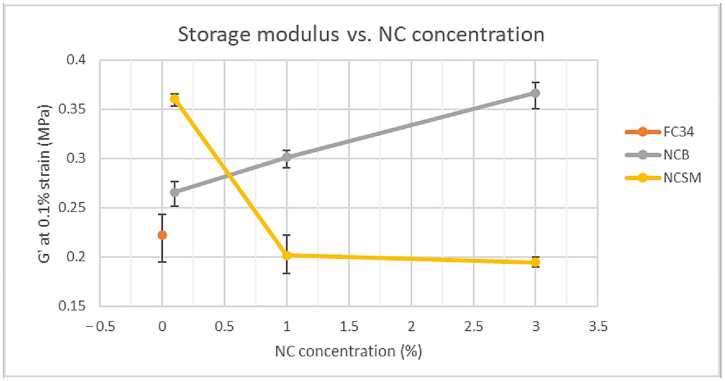
Comparison of NC-incorporated hydrogels storage modulus at 0.1% strain.

G′ at 0.1% strain was recorded for all samples, as all G′ curves preserved linearity up to at least 0.1% strain, indicating the gels stayed in their elastic state. These G′ values were then compared and are shown in Figure 8. NCSM had a detrimental effect on the F34 hydrogels stiffness except for a very small amount of NCSM, i.e., 0.1%. As explained in work conducted by Muralishwara et al., this could be due to a small percentage of NCSM being evenly distributed in the polymer matrix, causing the matrix’s strengthening properties and improved interfacial bonding [43]. However, hydrogels containing 1.0% and 3.0% of NCSM showed degraded G′ due to the wide agglomeration in the polymer matrix [44]. In addition, higher concentrations of NCSM could have interfered with polymer chain entanglements, which led to mechanical weakening. On the other hand, the incorporation of NCB showed a positive impact on the hydrogels G′. From Figure 8, the G′ of 3.0NCB_FC34 can be noticed to have risen approximately 65% from F34. Likewise, Müller et al. reported a much stronger starch-based film after incorporating 3% of NCB [45]. The rheological analysis proved that the mechanical performance of F34 was improved significantly by incorporating NCB or a small percentage of NCSM.

### 3.7. Scanning Electron Microscopy

**Figure 9:**
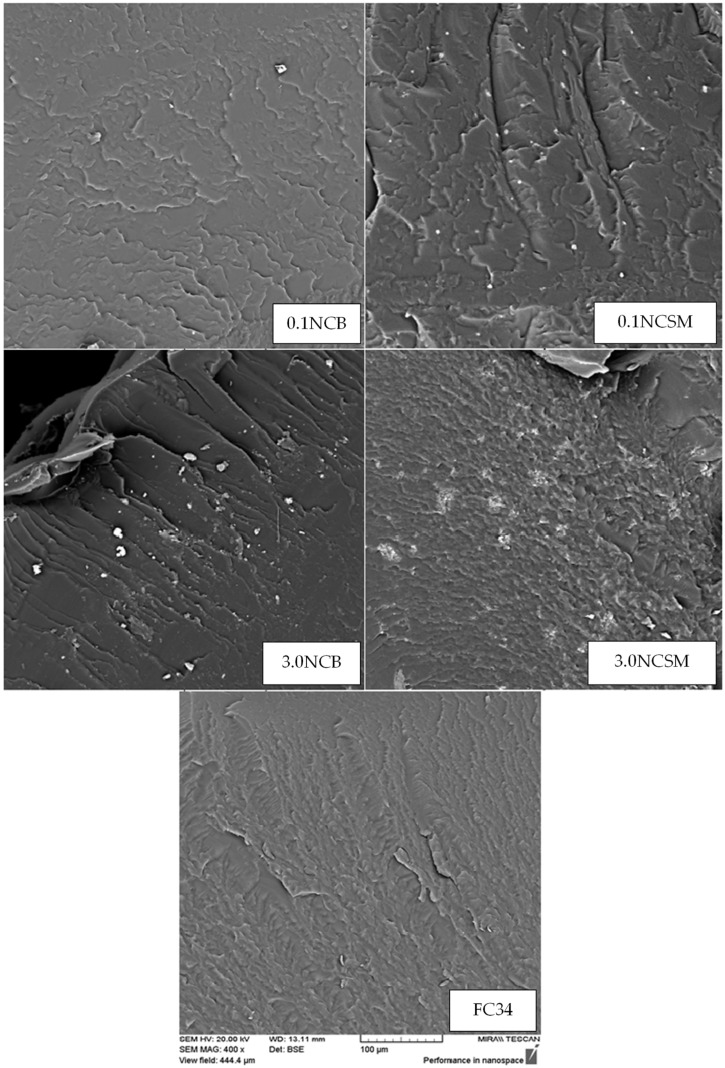
SEM images under 400× magnification with identical scale.

SEM is a technique used to detect the materials topography and surface morphology using a beam of high-energy electrons [46]. Prior to testing, the synthesised gels were soaked in liquid nitrogen and subsequently fractured with high-impact force. Figure 9 presents SEM images for FC34 and FC34 gels with 0.1% and 3.0% of NC (both NCB and NCSM). The hydrogels physical, chemical and biological properties can often be improved by the incorporation of nanoparticles [47]. From the conducted SEM, it can be observed that the applied fabrication method resulted in the discovery of traces of both NCB and NCSM particles within the gels. The SEM analysis shows a change in fracture patterns when incorporating different NC concentrations and types of NC in FC34. With 0.1% NCB and 0.1% NCSM, the gels started to develop clearer line-like fractures. Increasing the NCB concentration from 0.1% to 3.0% caused the gel to develop a parallel river-like fracture, which is comparable to other polymer resin composites [48]. This apparent crack propagation formed in the 3.0NCB_FC34 gel implies the strengthening of hydrogel polymer structure with NCB, which is caused by the better orientation of polymer chains. All NC-incorporated hydrogels were tested with improved storage modulus from the rheological analysis except those with 1.0% and 3.0% of NCSM. From the SEM image of 3.0NCSM_FC34 gel, it can be noticed that the surface fracture is more irregularly formed than FC34 gel. This phenomenon further justifies the weakening effect of NCSM on the gels G′ due to the wide agglomeration of NCSM at higher concentrations [44]. While 0.1NCSM_FC34 has a comparable analysed G’ with 3.0NCB_FC34, it shows a different fracture surface by having wave-like crack propagation. Both patterns of crack propagation in 0.1NCSM_FC34 and 3.0NCB_FC34 gels follow one direction, and this explains their higher G′ value compared to the others due to better orientation of polymer chains in their matrix.

## 4. Conclusions

NC-incorporated NVCL-NIPAm copolymers were successfully synthesised through UV irradiation, which was proven by the FTIR results. The cloud-point analysis and UV spectroscopy demonstrated the LCST of the physically crosslinked hydrogels were not affected by incorporating any type of NC between 0.1 and 3.0% of concentration. From the DOE, the significance of variables computed in descending order was the temperature, types of NC, and NC concentration, which were all found to be statistically significant in yielding the hydrogels swelling ratio. The 2-way and 3-way interactions between all variables were also found statistically significant, except the interaction between the NC concentration and temperature. The regression equation established through an R-squared value of 99.61% can be used to predict the hydrogels’ swelling ratio when the variables align with the equation parameter settings. The 3.0NCB_FC34 gel was found to be the most stable formulation, retaining its structural integrity even after 4.5 cycles of pulsatile swelling between 20 and 50 °C. Although some distortion of structural integrity was still observed, further studies, such as integrating different nanoparticles and fibrous materials like cellulose or silk fibres, have the potential to further improve the structural integrity of this hydrogel during pulsatile swelling. Nevertheless, 3.0NCB_FC34 gel has better swelling ability when compared with FC34 and 3.0NCSM_FC34 gels. From the rheological analysis, NC-incorporated gels have shown increased G’ than FC34 gel, except at 1.0 and 3.0% of NCSM. The SEM images explained the good distribution of NC particles in the gels and the great improvement in polymer orientation at 3.0% of NCB, yet at higher NCSM percentages, irregular polymer orientation was observed. Overall, FC34 gel swelling ability and mechanical strength were enhanced through the incorporation of NCB at a high percentage and NCSM at a low percentage. These findings have fundamental implications for the development of NC-incorporated NVCL-NIPAm hydrogels. Future work will involve utilising the developed formulation to create smart, shapeshifting prototypes tailored for certain applications.

## Figures and Tables

**Table 1:** The materials used in the study.

Materials	Chemical Structures or Composition
N-vinylcaprolactam (NVCL)	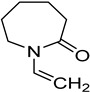
N-isopropylacrylamide (NIPAm)	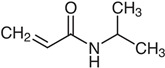
Poly(ethylene glycol) dimethacrylate (PEGDMA)	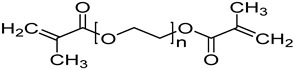
4-(2-hydroxyethoxy) phenyl-(2-hydroxy-2-propyl) ketone (Irgacure 2959)	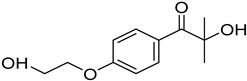
Bentonite clay	Al_2_H_2_O_6_Si

**Table 2:** Chemically crosslinked xerogels composition.

	Monomers		Crosslinker	Photoinitiator	Additives	
Formulation	NVCL (wt%)	NIPAm (wt%)	PEGDMA (wt%)	Irgacure 2959 (wt%)	NCSM (wt%)	NCB (wt%)
FC34	59.94	29.97	9.99	0.10	-	-
0.1NCB_FC34	59.94	29.97	9.99	0.10	-	0.1
1.0NCB_FC34	59.94	29.97	9.99	0.10	-	1.0
3.0NCB_FC34	59.94	29.97	9.99	0.10	-	3.0
0.1NCSM_FC34	59.94	29.97	9.99	0.10	0.1	-
1.0NCSM_FC34	59.94	29.97	9.99	0.10	1.0	-
3.0NCSM_FC34	59.94	29.97	9.99	0.10	3.0	-

**Table 3:** Physically crosslinked xerogels composition.

	Monomers		Photoinitiator	Additives	
Formulation	NVCL (wt%)	NIPAm (wt%)	Irgacure 2959 (wt%)	NCSM (wt%)	NCB (wt%)
FP34	66.60	33.30	0.10	-	-
0.1NCB_FP34	66.60	33.30	0.10	-	0.1
1.0NCB_FP34	66.60	33.30	0.10	-	1.0
3.0NCB_FP34	66.60	33.30	0.10	-	3.0
0.1NCSM_FP34	66.60	33.30	0.10	0.1	-
1.0NCSM_FP34	66.60	33.30	0.10	1.0	-
3.0NCSM_FP34	66.60	33.30	0.10	3.0	-

**Table 4:** DOE factors and configurations.

Factors	Levels	Values
NC types	2	NCB, NCSM
Percentages of NC (%)	3	0.1, 1.0, 3.0
Swelling temperature (°C)	2	20, 50

**Table 5:** DOE runs and their swelling result.

StdOrder	Types of NC	Concentration (%)	Temperature (°C)	Swelling Ratio (%)
1	NCB	0.1	20	150
2	NCB	0.1	50	48
3	NCSM	0.1	20	149
4	NCSM	0.1	50	30
5	NCB	1.0	20	158
6	NCB	1.0	50	41
7	NCSM	1.0	20	118
8	NCSM	1.0	50	26
9	NCB	3.0	20	160
10	NCB	3.0	50	42
11	NCSM	3.0	20	126
12	NCSM	3.0	50	23
13	NCB	0.1	20	142
14	NCB	0.1	50	43
15	NCSM	0.1	20	155
16	NCSM	0.1	50	35
17	NCB	1.0	20	160
18	NCB	1.0	50	40
19	NCSM	1.0	20	129
20	NCSM	1.0	50	28
21	NCB	3.0	20	160
22	NCB	3.0	50	41
23	NCSM	3.0	20	111
24	NCSM	3.0	50	22
25	NCB	0.1	20	147
26	NCB	0.1	50	34
27	NCSM	0.1	20	155
28	NCSM	0.1	50	37
29	NCB	1.0	20	154
30	NCB	1.0	50	39
31	NCSM	1.0	20	127
32	NCSM	1.0	50	27
33	NCB	3.0	20	156
34	NCB	3.0	50	39
35	NCSM	3.0	20	109
36	NCSM	3.0	50	20

**Table 6:** LCST determined from both cloud-point analysis and UV spectroscopy.

Formulation	Cloud-Point (°C)	UV Spectroscopy (°C)
FP34	32	31
0.1NCB_FP34	32	31
1.0NCB_FP34	32	31
3.0NCB_FP34	32	31
0.1NCSM_FP34	32	31
1.0NCSM_FP34	32	31
3.0NCSM_FP34	32	31

## Data Availability

Data are contained within the article.

## Appendix A

**Table A1:** Model summary for the conducted DOE.

S	R-Sq	R-Sq (Adj)	R-Sq (Pred)
4.43613	99.61%	99.39%	98.97%

Swelling Ratio = 88.361 + 9.083 NC_NCB − 9.083 NC_NCSM + 5.39 Conc._0.1 − 1.11 Conc._1.0 − 4.28 Conc._3.0 + 54.194 Temp._20 − 54.194 Temp._50 − 8.83 NC × Conc._NCB 0.1 + 2.33 NC × Conc._NCB 1.0 + 6.50 NC × Conc._NCB 3.0 + 8.83 NC × Conc._NCSM 0.1 − 2.33 NC × Conc._NCSM 1.0 − 6.50 NC × Conc._NCSM 3.0 + 2.472 NC × Temp._NCB 20 − 2.472 NC × Temp._NCB 50 − 2.472 NC × Temp._NCSM 20 + 2.472 NC × Temp._NCSM 50 + 1.72 Conc. × Temp._0.1 20 − 1.72 Conc. × Temp._0.1 50 − 0.44 Conc. × Temp._1.0 20 + 0.44 Conc. × Temp._1.0 50 − 1.28 Conc. × Temp._3.0 20 + 1.28 Conc. × Temp._3.0 50 − 6.06 NC × Conc. × Temp._NCB 0.1 20 + 6.06 NC × Conc. × Temp._NCB 0.1 50 + 2.44 NC × Conc. × Temp._NCB 1.0 20 − 2.44 NC × Conc. × Temp._NCB 1.0 50 + 3.61 NC × Conc. × Temp._NCB 3.0 20 − 3.61 NC × Conc. × Temp._NCB 3.0 50 + 6.06 NC × Conc. × Temp._NCSM 0.1 20 − 6.06 NC × Conc. × Temp._NCSM 0.1 50 − 2.44 NC × Conc. × Temp._NCSM 1.0 20 + 2.44 NC × Conc. × Temp._NCSM 1.0 50 − 3.61 NC × Conc. × Temp._NCSM 3.0 20 + 3.61 NC × Conc. × Temp._NCSM 3.0 50 (A1)

**Table A2:** ANOVA table for the conducted DOE. Interaction terms are denoted by inserting an asterisk between the variables intended for interaction.

Source	DF	Adj SS	Adj MS	F-Value	*p*-Value
Model	13	111,775	8598	436.91	0.000
Blocks	2	34	17	0.87	0.431
Linear	4	109,286	27,322	1388.34	0.000
Types of NC	1	2970	2970	150.93	0.000
Concentration (%)	2	583	291	14.81	0.000
Temperature	1	105,733	105,733	5372.82	0.000
2-Way Interactions	5	1786	357	18.15	0.000
Types of NC*Concentration (%)	2	1509	754	38.33	0.000
Types of NC*Temperature	1	220	220	11.18	0.003
Concentration (%)*Temperature	2	58	29	1.46	0.253
3-Way Interactions	2	668	334	16.98	0.000
Types of NC*Concentration (%)*Temperature	2	668	334	16.98	0.000
Error	22	433	20		
Total	35	112,208			
